# Alleviation of Oxidative Damage and Involvement of Nrf2-ARE Pathway in Mesodopaminergic System and Hippocampus of Status Epilepticus Rats Pretreated by Intranasal Pentoxifylline

**DOI:** 10.1155/2017/7908072

**Published:** 2017-03-12

**Authors:** Yunxiao Kang, Wensheng Yan, Hui Fang, Guoliang Zhang, Yakun Du, Lei Wang, Huixian Cui, Geming Shi

**Affiliations:** ^1^Department of Neurobiology, Hebei Medical University, Shijiazhuang, Hebei 050017, China; ^2^Department of Human Anatomy, Shijiazhuang Medical College, Shijiazhuang, Hebei 050599, China; ^3^Department of Human Anatomy, Hebei Medical University, Shijiazhuang, Hebei 050017, China; ^4^Department of Neurology, Children's Hospital of Hebei Province, Shijiazhuang, Hebei 050017, China

## Abstract

The current studies were aimed at evaluating the efficacy of intranasal pentoxifylline (Ptx) pretreatment in protecting mesodopaminergic system and hippocampus from oxidative damage of lithium-pilocarpine induced status epilepticus (SE) and the involvement of nuclear factor erythroid 2-related factor 2- (Nrf2-) antioxidant response elements pathway. Pentoxifylline was administered to rats intranasally or intraperitoneally 30 minutes before inducing SE. Our results showed the impaired visuospatial memory, the defected mesodopaminergic system, and the oxidative damage and the transient activation of Nrf2 in SE rats. The transient activation of Nrf2 in SE rats was enhanced by Ptx pretreatment, which was followed by the upregulation of heme oxygenase-1 and NAD(P)H:quinone oxidoreductase-1. Ptx pretreatment to SE rats significantly suppressed the epileptic seizures, decreased the levels of lipid peroxide and malondialdehyde, and elevated the ratio of reduced glutathione/oxidized glutathione. Compared with intraperitoneal injection, intranasal Ptx delivery completely restored the visuospatial memory and the activity of mesodopaminergic system in SE rats. Intranasal administration of Ptx may hopefully become a noninvasive, painless, and easily administered option for epileptic patients.

## 1. Introduction

Epilepsy is a life-threatening medical emergency that warrants immediate treatment to prevent seizure activity and associated neuronal damage [1]. Status epilepticus (SE) is one of the most severe conditions of epilepsy [2]. Patients with SE are at risk of neurologic complications. Early intervention is frequently needed to shorten seizure duration [3]. The sooner seizures are treated, the more likely they will be controlled [4]. However, intramuscular or intravenous injection cannot be implemented immediately outside of hospital settings, thus delaying or missing the best treatment time and threatening the human life. The intranasal route to administer drugs is quick and effective in targeting the brain [4, 5] and potentially provides a direct delivery of the drug to the central nervous system [6, 7] bypassing the blood-brain barrier and exerting therapeutic effects at terminating epileptic seizures. Intranasal drug administration is noninvasive, painless, and easily administered for epileptic patients [4] in the home treatment of prolonged seizures and in the treatment of prehospital seizures by emergency medical technicians [8].

The first-line drugs to stop seizures are benzodiazepines [9], which manipulate *γ*-aminobutyric acid receptors and make neurons resistant to excitation. In addition to controlling neuronal hyperactivity and excitotoxicity, one important factor that should be dealt with is oxidative stress in epileptic seizures [10–12]. Oxidative stress means an imbalance between oxidation and antioxidation in vivo, which leads to excessive oxygen free radical and reduced antioxidative capacity [13–15]. Excessive oxygen free radical generated in SE dramatically impairs the structure and function of neurons [16–18]. Nuclear factor erythroid 2-related factor 2 (Nrf2), as a transcription factor, controls the basal and inducible expression of an array of antioxidant and detoxification enzymes to degrade oxygen free radical [13, 19, 20]. Disruption of Nrf2-antioxidant response elements (ARE) pathway results in an increased susceptibility to oxidative insults and other toxicants [21]. Activation of Nrf2-ARE pathway protects neurons against oxidative damage and excitotoxic damage [13, 22–24].

Pentoxifylline (Ptx), a potent antioxidant and modulator of a variety of transmitters, was initially introduced for the treatment of respiratory and peripheral circulatory disorders. Currently, a beneficial effect of Ptx is found on preventing epileptic seizures [25, 26]. The frequency and severity of epileptic seizures are ameliorated and oxidative damage is attenuated in Ptx-treated SE rats [25, 26]. Clinical and experimental studies have suggested the implication of dopaminergic system in seizures [27], such as a decreased DA content in hippocampus (Hip) and striatum of SE rats [26, 28], as well as the reduced dopamine transporter (DAT) in substantia nigra (SN) and midbrain of epilepsy patients [29, 30]. Therefore, in the present study, the efficacy of intranasal Ptx delivery in epileptic seizures was investigated by analyzing the alteration of mesodopaminergic system and hippocampus in SE rats induced by lithium-pilocarpine (Li-Pc), based on the severe implication of mesodopaminergic system and Hip in SE [26, 29]. Meanwhile, oxidative stress parameters and Nrf2-ARE pathway were examined to evaluate whether Nrf2 was involved in the effects of Ptx treatment on mesodopaminergic system and hippocampus of SE rats. For comparison purposes, the same observations were performed in SE rats that experienced intraperitoneal Ptx treatment.

## 2. Materials and Methods

### 2.1. Animals and Housing

Three-month-old male Sprague Dawley rats were supplied by the Experimental Animal Center of Hebei Medical University and were housed under controlled conditions with 12-hour light-dark diurnal cycle at 22 ± 2°C, with humidity at 50–60% and with free access to food and water. The experimental procedures followed the rules in the “Guidelines for the Care and Use of Mammals in Neuroscience and Behavioral Research” and were approved by the Committee of Ethics on Animal Experiments at Hebei Medical University.

### 2.2. Ptx Treatment and Induction of SE

Ptx was administered to rats either via nostril instillation or by intraperitoneal injection. For intranasal experiment, experimental rats were assigned to the following groups: CON-in (*n* = 25), LICI-in (*n* = 25), PTX-in (*n* = 25), SE-in (*n* = 35), and PTX.in-SE (*n* = 35). The rats in PTX-in and PTX.in-SE were intranasally given Ptx. Intranasal Ptx delivery was performed as described [31]. Briefly, fully conscious rats were held and laid upside down. The solution of Ptx (prepared in saline) was introduced by the pressure with a micropipette into one nasal cavity, without introducing the pipette tip directly into the nasal cavity, and the rats were immobilized in this position for 15 s by gently pulling the tail to prevent sneezing. The rats were discarded if sneezing happened. The same procedure was repeated in the other nostril. For intraperitoneal injection experiment, rats were divided into five groups consisting of CON-ip (*n* = 25), LICI-ip (*n* = 25), PTX-ip (*n* = 25), SE-ip (*n* = 35), and PTX.ip-SE (*n* = 35). The rats in PTX-ip and PTX.ip-SE received intraperitoneal Ptx injection. SE was induced in SE-in, SE-ip, PTX.in-SE, and PTX.ip-SE rats by administering an aqueous solution of lithium chloride (Li, 127 mg/kg, BDH Laboratory Supplies) intraperitoneally, followed (20 hrs later) by injecting pilocarpine hydrochloride (Pc, 20 mg/kg, Sigma) subcutaneously. Ptx was administered intranasally or intraperitoneally at the dose of 60 mg/kg (30 minutes before Pc injection) to rats corresponding to PTX.in-SE and PTX.ip-SE, respectively. The rats in CON-in, LICI-in, and PTX-in as well as CON-ip, LICI-ip, and PTX-ip received saline, lithium chloride, and Ptx (60 mg/kg, Sigma) correspondingly. After Pc injections, the rats were observed for the signs of seizure activity. Based on Racine's scale [32], the rats that showed consecutive seizures with a score of 3 or above fell in SE [25, 32]. The latency and incidence of seizures as well as mortality within 24 hrs were recorded. The rats were sacrificed 24 hrs (for oxidative parameters and Nrf2-ARE pathway) or 7 days (for Morris water maze test and parameters of mesodopaminergic system as well as oxidative parameters and Nrf2-ARE pathway) following Pc treatment. For the rats that were treated similarly in the groups CON-in (and ip), LICL-in (and ip), or SE-in (and ip), since the parameters were not substantially different (ANOVA), the data collected from them in the two experiments were clustered (cl) for analysis as CON-cl, LICL-cl, or SE-cl correspondingly, except for the data collected by Western blot.

### 2.3. Morris Water Maze Test

The rats in each group were tested for visuospatial memory 24 hrs after the Pc injection using Morris water maze test as described previously [24]. The water maze included a circular water tank (180 cm in diameter, 80 cm high) that was partially filled with water (23 ± 1°C). The water was made opaque by adding milk to prevent visualization of the platform. The pool was divided virtually into four equal quadrants. A colorless escape platform (10 cm in diameter) was hidden 1 cm below the surface of the water in a fixed location. The maze was located in a quiet room, surrounded by visual cues outside of the maze, which was used by the rats for spatial orientation. The experiments were conducted two sessions per day for 5 consecutive days, each session including four trials, with an intertrial interval of 60 s and an intersession interval of 2 hrs. In each trial, the animals were gently placed in the middle of the circular edge in a randomly selected quadrant, with the nose pointing toward the wall. If animals failed to find the escape platform within 120 s by themselves, they were placed on the platform for 10 s by the experimenter and their escape latency was accepted as 120 s. After climbing onto the platform, the animal remained there for 30 s before the commencement of the next trial. On the sixth day, a probe trial without the platform was assessed, and the time spent in the target quadrant where the platform had been located was recorded.

### 2.4. Sample Preparation

For biochemical, real-time quantitative PCR (qPCR) and Western blot analyses, the rats in each group were sacrificed by decapitation. The brains were removed quickly. The tissue block containing substantia nigra and ventral tegmental area (SN-VTA; between 3.00 mm and 4.08 mm) or caudate putamen (CPu; between 8.64 mm and 10.08 mm) and Hip (between 5.40 mm and 6.08 mm) relatively rostral to the interaural axis was dissected on ice-cold plate using a scalpel for ophthalmic surgery and a stereomicroscopy. The tissue blocks of rats in each group were processed for Western blot or liquid chromatography coupled with tandem mass spectrometry (LC-MS/MS) assay and the tissue blocks of others were chosen for lipid peroxide (LPO), malondialdehyde (MDA), reduced glutathione (GSH), and oxidized glutathione (GSSG) assay by spectrophotometry or prepared for qPCR analysis based on the study purposes.

### 2.5. Biochemical Analysis

For LPO, MDA, GSH, and GSSG assay, SN-VTA or Hip tissue block was weighed and homogenized with 10 times (w/v) ice-cold 0.1 M phosphate buffer, PH 7.4. The homogenates were used to assess LPO, MDA, GSH, and GSSG spectrophotometrically using detection kits following the manufacturer's instruction (Nanjing Jiancheng Bioengineering Institute, China).

For dopamine (DA) and metabolites assay, CPu or Hip tissue block was weighed and homogenized in 80% acetonitrile containing 0.1% formic acid (5 *μ*L/mg). The homogenates were centrifuged at 14,000 rpm for 10 min at 4°C. The supernatants were collected and stored at −80°C. DA, 3,4-dihydroxyphenylacetic acid (DOPAC), and homovanillic acid (HVA) were determined by LC-MS/MS. The LC separation was performed on Agilent 1200 LC system (Agilent, Santa Clara, USA) using a Synergi Fusion-RP C18 column (50 mm × 3.0 mm, 4 *μ*m) provided by Phenomenex. MS/MS detection was carried out using a 3200 QTRAP™ LC-MS/MS System (Applied Biosystems, Foster City, CA, USA). The multiple-reaction monitoring mode was used for the quantification. The principal validation parameters of the LC-MS/MS were set up as shown in Table 1, based on the previous study [33].

### 2.6. qPCR

2 *μ*g of total RNA from the SN-VTA or Hip tissue block was subjected to reverse transcription using random primer to obtain the first-strand cDNA template. qPCR was performed with 0.8 *μ*L cDNA (diluted 1 : 10), specific primers 2 *μ*L, and 2 × GoTaq® Green Master Mix (Promega, USA) with a final volume of 20 *μ*L. PCR was performed as follows: an initial cycle at 95°C for 10 min, followed by 40 cycles at 95°C for 15 s, 58°C for 20 s, and 72°C for 27 s. Then PCR products were analyzed by melting curve to confirm the specificity of amplification. Expression of tyrosine hydroxylase (TH), dopamine transporter (DAT), Nrf2, heme oxygenase-1 (HO-1) and NAD(P)H:quinone oxidoreductase-1 (NQO-1) genes was detected. The relative quantification was calculated using the 2^−ΔΔct^ method. GAPDH was used as reference gene in all calculations. The sets of primers were as follows: TH (5′-GCTTCTCTGACCAGGTGTATCG-3′ and 5′-GCAATCTCTTCCGCTGTGTAT-3′), DAT (5′-ACTCTGTGAGGCATCTGTGTG-3′ and 5′-TGTAACTGGAGAAGGCAATCAG-3′), Nrf2 (5′-GACCTAAAGCACAGCCAACACAT-3′ and 5′-CTCAATCGGCTTGAATGTTTGTC-3′), HO-1 (5′-TGTCCCAGGATTTGTCCGAG-3′ and 5′-ACTGGGTTCTGCTTGTTTCGCT-3), NQO-1 (5′-GGGGACATGAACGTCATTCTCT-3′ and 5′-AGTGGTGACTCCTCCCAGACAG-3′), and GAPDH (5′-TGAACGGGAAGCTCACTG-3′ and 5′-GCTTCACCACCTTCTTGATG-3′).

### 2.7. Western Blot Analysis

For detection of TH and DAT protein levels, SN-VTA, CPu, or Hip tissue block was homogenized in Radioimmunoprecipitation Assay (RIPA) buffer containing 1% Triton X-100, 0.1% SDS, 0.5% sodium deoxycholate, and protease inhibitors (phenylmethanesulfonyl fluoride 100 *μ*g/mL, aprotinin 30 *μ*g/mL, and sodium orthovanadate 1 mM) and then sonicated for 4 × 10 s. After centrifugation at 12,000 ×g for 20 min at 4°C, the supernatant was collected and centrifuged again as above. The final resulting supernatant was stored at −80°C until use. Samples from SN-VTA, CPu, or Hip were diluted in 2x sample buffer (50 mM Tris, pH 6.8, 2% SDS, 10% glycerol, 0.1% bromophenol blue, and 5%  *β*-mercaptoethanol) and heated for 5 min at 95°C before SDS-PAGE on a 10% gel and subsequently transferred to a PVDF membrane. The membrane was incubated for 2 h with 5% nonfat dry milk in Tris-buffered saline (TBS) containing 0.05% Tween-20 (TBST) (20 mM Tris–Cl, 137 mM NaCl, 0.1% Tween 20, pH 7.6) at room temperature. The membrane was rinsed in three changes of TBST and then incubated overnight with mouse anti-TH monoclonal antibody (1 : 10,000, Sigma) or rabbit anti-DAT polyclonal antibody (1 : 4000, Millipore) at 4°C. After three washes, the membrane was incubated for 1 h in IRDye® 800-conjugated goat anti-mouse second antibody (1 : 3000, Rockland) or goat anti-rabbit second antibody (1 : 3000, Rockland) at room temperature. The relative density of bands was analyzed on an Odyssey infrared scanner (LI-COR Biosciences). Following stripping, each PVDF membrane was subsequently immunoblotted with mouse anti-*β*-actin monoclonal antibody (1 : 6000, Santa Cruz Biotechnology). The labeling densities for TH or DAT were compared with those of *β*-actin, which were the endogenous control.

For detection of Nrf2, HO-1, or NQO-1 protein levels, SN-VTA or Hip tissue block was homogenized in ice-cold lysis buffer (10 mmol/L HEPES, pH 7.9, 10 mmol/L KCl, 0.1 mmol/L EDTA, 1 mmol/L DTT, and 0.1 mmol/L EGTA) for 15 min. After adding NP-40, the homogenate was centrifuged at 10,000 rpm at 4°C for 3 min and the supernatant was collected as cytoplasmic protein for HO-1 and NQO-1. The pellets were homogenized in ice-cold lysis buffer (20 mmol/L HEPES, pH 7.9, 400 mmol/L NaCl, 1 mmol/L EDTA, and 0.1 mmol/L EGTA) for 15 min. Then the pellets were centrifuged at 12,000 rpm at 4°C for 10 min, and the supernatant was collected. Phenylmethanesulfonyl fluoride was added to the supernatant with the final concentration of 1 mmol/L as the nuclear protein for Nrf2. Samples from SN-VTA or Hip (50 *μ*g) were separated by SDS/PAGE and transferred onto PVDF membranes. Membranes were blocked with 5% skimmed milk for 1 h at room temperature and then were probed with polyclonal rabbit anti-Nrf2 antibody (1 : 500, Abcam), polyclonal rabbit anti-HO-1 antibody (1 : 200, Abcam), or polyclonal rabbit anti-NQO-1 antibody (1 : 200, Abcam) overnight at 4°C. After washing three times with phosphate buffered saline with 1% Tween 20, IRDye 800-conjugated goat anti-rabbit second antibody (1 : 3000, Rockland) incubated with membranes for 1 h at room temperature. The relative density of bands was analyzed on an Odyssey infrared scanner (LI-COR Biosciences). The densitometry values were normalized with respect to the values of anti-histone 3 for Nrf2 or anti-*β*-actin for HO-1 and NQO-1 immunoreactivity.

### 2.8. Statistical Analysis

Measurement data were described with mean ± SD. Levene's test was applied to test homogeneity of variance. If both normal distribution (*P* > 0.1) and homogeneity of variance (*P* > 0.1) were found, then parametric test was performed by one-way analysis of variance (one-way ANOVA) followed by a Student-Newman-Keuls (SNK) post hoc test for multiple comparisons. Otherwise, nonparametric statistics were done by Kruskal-Wallis test followed by Mann–Whitney* U* test for post hoc analysis between groups. A difference was considered statistically significant at a *P* value of less than 0.05.

## 3. Results

### 3.1. Antiepileptic Effects of Ptx Pretreatment

The effects of intranasal Ptx pretreatment on epileptic activities were analyzed. All the rats in SE-cl developed into epileptic seizures and then into SE after the injection of Pc. 10% of them died over a period of 24 hrs. Ptx pretreatment significantly extended the latency to first seizure and decreased epileptic seizures. 17.14% of rats in PTX.in-SE and 20% of rats in PTX.ip-SE developed into seizures. No rats in PTX.in-SE and PTX.ip-SE developed into SE and died (Table 2). Intranasal Ptx administration lessened epileptic activities as intraperitoneal injection of Ptx did and had antiepileptic effects.

### 3.2. Visuospatial Memory

**Figure 1:**
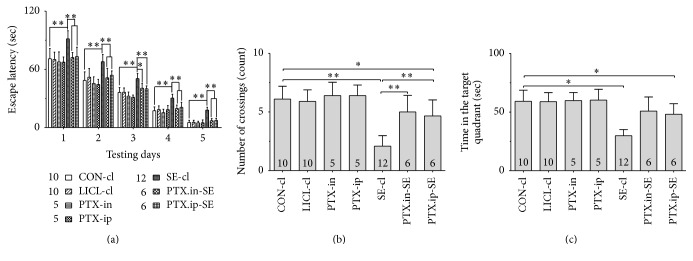
Effects of intranasal and intraperitoneal Ptx pretreatment on the visuospatial memory of SE rats induced by Li-Pc. (a) The escape latency to reach the platform. (b) The number of crossings. (c) The time spent in the target quadrant. The results were expressed as the means ± SD. ^*∗*^*P* < 0.05; ^*∗∗*^*P* < 0.01.

Behavioral parameters in water maze test were analyzed to reveal the effects of intranasal Ptx pretreatment on visuospatial memory of SE rats. Group differences in the escape latency (Figure 1(a), 1 d: *χ*^2^ = 23.338 and *P* < 0.01; 2 d: *χ*^2^ = 26.167 and *P* < 0.01; 3 d: *F*(6,47) = 17.824 and *P* < 0.01; 4 d: *χ*^2^ = 30.381 and *P* < 0.01; 5 d: *χ*^2^ = 32.735 and *P* < 0.01), the number of crossings (Figure 1(b), *χ*^2^ = 33.250 and *P* < 0.01), and the time in the target quadrant (Figure 1(c), *χ*^2^ = 32.996 and *P* < 0.01) were found among the CON-cl, LICL-cl, PTX-in, PTX-ip, SE-cl, PTX.in-SE, and PTX.ip-SE rats. The post hoc test showed that the rats in SE-cl exhibited the longer escape latency to reach the platform (*P* < 0.01), the reduced number of crossings (*P* < 0.01), and the less time in the target quadrant (*P* < 0.05), compared with rats in CON-cl. Intranasal or intraperitoneal Ptx pretreatment to SE rats shortened the escape latency to reach the platform (*P* < 0.05) and increased the number of crossings and the time spent in the target quadrant (*P* < 0.01). There were no differences in the behavioral parameters of rats between PTX.in-SE and PTX.ip-SE. The behavioral parameters of rats in PTX.in-SE were completely restored to CON-cl rats, compared with rats in PTX.ip-SE. Intranasal Ptx pretreatment ameliorated the poor visuospatial memory of SE rats.

### 3.3. Mesodopaminergic System

To reveal whether intranasal Ptx pretreatment ameliorated the mesodopaminergic activity in SE rats, the markers of mesodopaminergic system were analyzed.

#### 3.3.1. DA and Its Metabolites

Group differences among CON-cl, LICL-cl, PTX-in, PTX-ip, SE-cl, PTX.in-SE, and PTX.ip-SE rats were observed in DA (CPu: *χ*^2^ = 37.676 and *P* < 0.01; Hip: *F*(6,47) = 78.847 and *P* < 0.01), DOPAC (CPu: *F*(6,47) = 45.800 and *P* < 0.01; Hip: *F*(6,47) = 146.455 and *P* < 0.01), and HVA content (CPu: *F*(6,47) = 73.834 and *P* < 0.01; Hip: *F*(6,47) = 55.916 and *P* < 0.01) (Table 3). The post hoc test revealed the decreased DA, DOPAC, and HVA in CPu and Hip of rats in SE-cl, compared with rats in CON-cl (*P* < 0.01). Intranasal or intraperitoneal Ptx pretreatment to SE rats increased DA and its metabolites in CPu and Hip (*P* < 0.01). No differences in DA and its metabolites were shown in CPu and Hip of rats between PTX.in-SE and PTX.ip-SE. Intranasal Ptx pretreatment completely restored DA and its metabolites of SE rats to the levels of CON-cl rats, compared with rats in PTX.ip-SE.

#### 3.3.2. TH and DAT mRNAs

**Figure 2:**
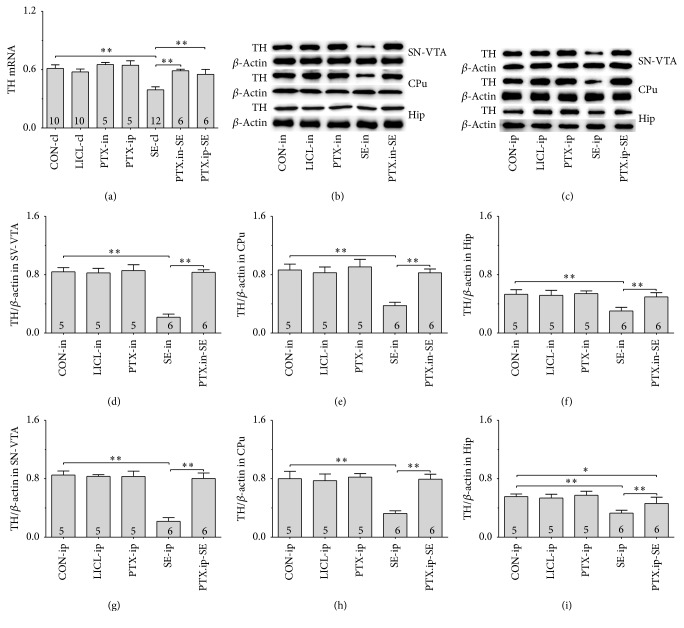
Effects of intranasal and intraperitoneal Ptx pretreatment on TH at mRNA and protein level of SE rats induced by Li-Pc. (a) TH mRNAs were detected in SN-VTA by qPCR. (b–i) TH protein was detected in SN-VTA, CPu, and Hip by Western blot. The results were expressed as the means ± SD. ^*∗*^*P* < 0.05; ^*∗∗*^*P* < 0.01.

**Figure 3:**
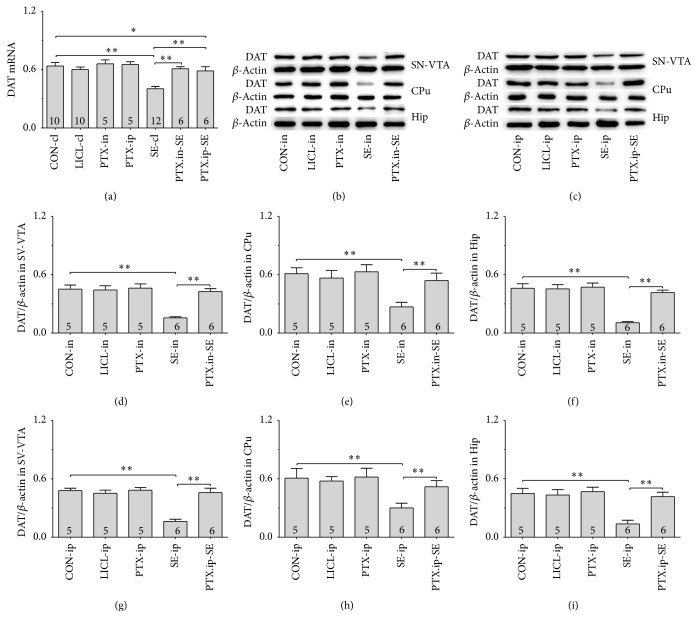
Effects of intranasal and intraperitoneal Ptx pretreatment on DAT at mRNA and protein level of SE rats induced by Li-Pc. (a) DAT mRNA was detected in SN-VTA by qPCR. (b–i) DAT protein was detected in SN-VTA, CPu, and Hip by Western blot. The results were expressed as the means ± SD. ^*∗*^*P* < 0.05; ^*∗∗*^*P* < 0.01.

Group differences in TH mRNA (Figure 2(a), *F*(6,47) = 62.738 and *P* < 0.01) and DAT mRNA (Figure 3(a), *χ*^2^ = 37.814 and *P* < 0.01) were detected in SN-VTA of rats among CON-cl, LICL-cl, PTX-in, PTX-ip, SE-cl, PTX.in-SE, and PTX.ip-SE. The post hoc test found that the levels of TH and DAT mRNAs were lower in SE-cl rats than in CON-cl rats (*P* < 0.01). Intranasal or intraperitoneal Ptx pretreatment to SE rats significantly increased TH and DAT mRNAs in SN-VTA (*P* < 0.01). No differences in TH and DAT mRNAs were shown in SN-VTA between PTX.in-SE rats and PTX.ip-SE rats. Intranasal Ptx pretreatment completely restored TH and DAT mRNAs of SE rats to the levels of CON-cl rats, compared with rats in PTX.ip-SE.

#### 3.3.3. TH and DAT Proteins

Western blot was used to reveal the protein levels of TH and DAT extracted from SN-VTA, CPu, and Hip. TH and DAT were located at approximately 60 and 80 kDa, respectively. The group differences among CON-in, LICL-in, PTX-in, SE-in, and PTX.in-SE rats were found in the expression of TH (Figures 2(b) and 2(d), SN-VTA: *F*(4,22) = 144.914 and *P* < 0.01; Figure 2(e), CPu: *F*(4,22) = 50.721 and *P* < 0.01; Figure 2(f), Hip: *F*(4,22) = 18.219 and *P* < 0.01) and DAT (Figures 3(b) and 3(d), SN-VTA: *χ*^2^ = 14.314 and *P* < 0.01; Figure 3(e), CPu: *χ*^2^ = 16.289 and *P* < 0.01; Figure 3(f), Hip: *χ*^2^ = 17.015 and *P* < 0.01), as well as in that of TH (Figures 2(c), 2(g)–2(i); SN-VTA, CPu, and Hip: *P* < 0.01) and DAT (Figures 3(c), 3(g)–3(i); SN-VTA, CPu, and Hip: *P* < 0.01) among CON-ip, LICL-ip, PTX-ip, SE-ip, and PTX.ip-SE rats. The post hoc test revealed the decreased TH and DAT in SE-in rats and in SE-ip rats, compared with corresponding control rats (*P* < 0.01). Intranasal delivery, as well as intraperitoneal injection, of Ptx to SE rats elevated the levels of TH and DAT in SN-VTA, CPu, and Hip (*P* < 0.01). There were no differences in the levels of TH and DAT between PTX.in-SE rats and PTX.ip-SE rats. Intranasal Ptx pretreatment to SE rats completely restored TH and DAT proteins in SN-VTA, CPu, and Hip, compared with rats in PTX.ip-SE.

### 3.4. LPO, MDA, GSH, and GSSG

**Figure 4:**
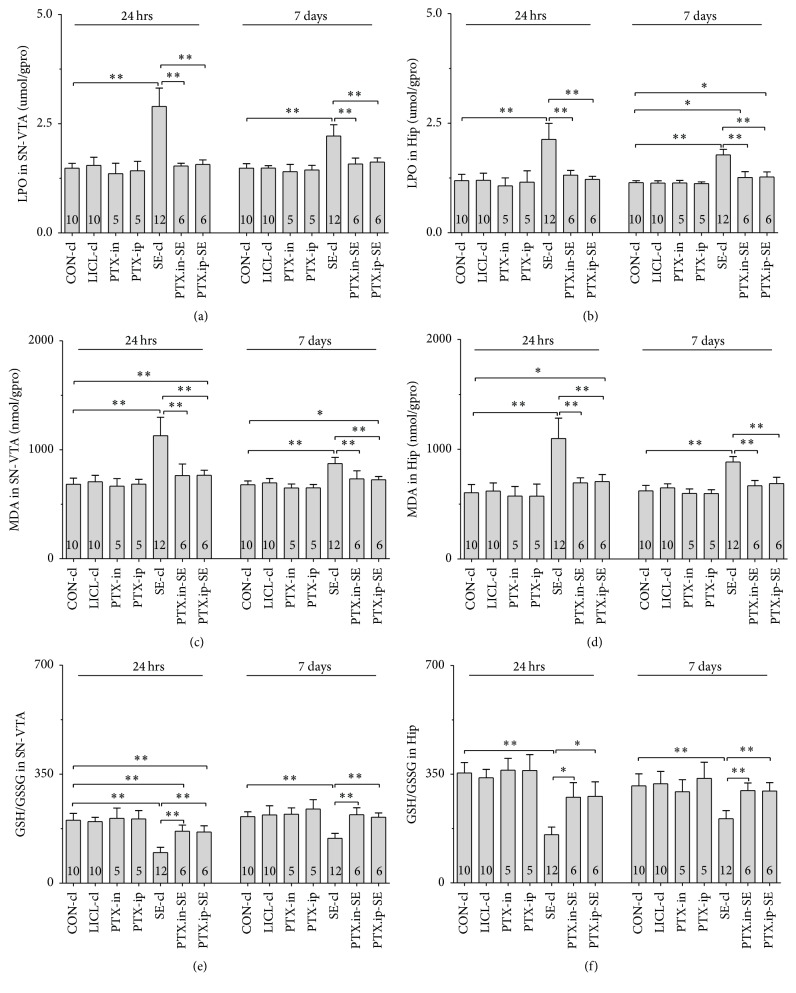
Effects of intranasal and intraperitoneal Ptx pretreatment on oxidative damage in SN-VTA and Hip of SE rats induced by Li-Pc 24 hrs and 7 days after Pc injection. (a) LPO in SN-VTA. (b) LPO in Hip. (c) MDA in SN-VTA. (d) MDA in Hip. (e) GSH/GSSG in SN-VTA. (f) GSH/GSSG in Hip. The results were expressed as the means ± SD. ^*∗*^*P* < 0.05; ^*∗∗*^*P* < 0.01.

To assess whether the amelioratory effects of intranasal Ptx pretreatment to SE rats was associated with the oxidative stress, LPO, MDA, and the ratio of GSH/GSSG in SN-VTA and Hip were analyzed. There were group differences in LPO (Figure 4(a), SN-VTA (24 hrs): *χ*^2^ = 32.447 and *P* < 0.01; SN-VTA (7 days): *χ*^2^ = 33.837 and *P* < 0.01; Figure 4(b), Hip (24 hrs): *χ*^2^ = 31.413 and *P* < 0.01; Hip (7 days): *χ*^2^ = 36.309 and *P* < 0.01), MDA (Figure 4(c), SN-VTA (24 hrs): *χ*^2^ = 33.813 and *P* < 0.01; SN-VTA (7 days): *χ*^2^ = 35.623 and *P* < 0.01; Figure 4(d), Hip (24 hrs): *χ*^2^ = 36.381 and *P* < 0.01; Hip (7 days): *χ*^2^ = 26.853 and *P* < 0.01), and the ratio of GSH/GSSG (Figure 4(e), SN-VTA (24 hrs): *χ*^2^ = 38.609 and *P* < 0.01; SN-VTA (7 days): *χ*^2^ = 30.277 and *P* < 0.01; Figure 4(f), Hip (24 hrs): *F*(6,47) = 42.160 and *P* < 0.01; Hip (7 days): *F*(6,47) = 13.550 and *P* < 0.01) among the CON-cl, LICL-cl, PTX-in, PTX-ip, SE-cl, PTX.in-SE, and PTX.ip-SE rats. The post hoc test detected the increased LPO and MDA contents as well as the decreased ratio of GSH/GSSG in SN-VTA and Hip of SE-cl rats, compared with CON-cl rats (*P* < 0.01). The reduced levels of LPO and MDA and the elevated ratio of GSH/GSSG in SN-VTA and Hip were detected in rats of PTX.in-SE as well as PTX.ip-SE, compared with the rats in SE-cl (*P* < 0.01). Intranasal Ptx pretreatment to SE rats mitigated the oxidative damage in SN-VTA and Hip.

### 3.5. Nrf2-ARE

To evaluate whether the Nrf2-ARE pathway was involved in the alleviation of the oxidative damage in SN-VTA and Hip of intranasal Ptx-pretreated SE rats, Nrf2, HO-1, and NQO-1 were examined at mRNA and protein levels.

#### 3.5.1. Nrf2, HO-1 and NQO-1 mRNAs

**Figure 5:**
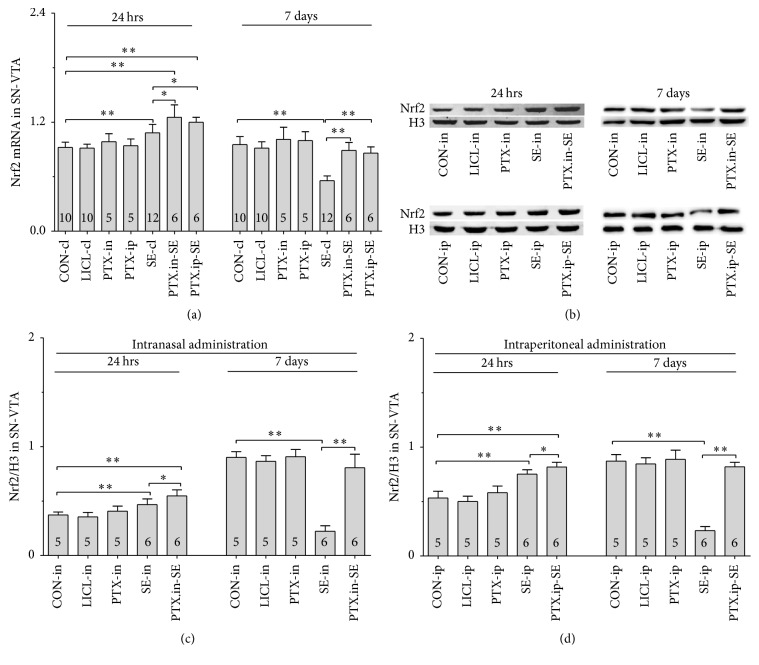
Effects of intranasal and intraperitoneal Ptx pretreatment on Nrf2 in SN-VTA of SE rats induced by Li-Pc 24 hrs and 7 days after Pc injection. (a) Nrf2 mRNA was detected by qPCR. (b–d) Nrf2 protein was measured by Western blot. The results were expressed as the means ± SD. ^*∗*^*P* < 0.05; ^*∗∗*^*P* < 0.01.

**Figure 6:**
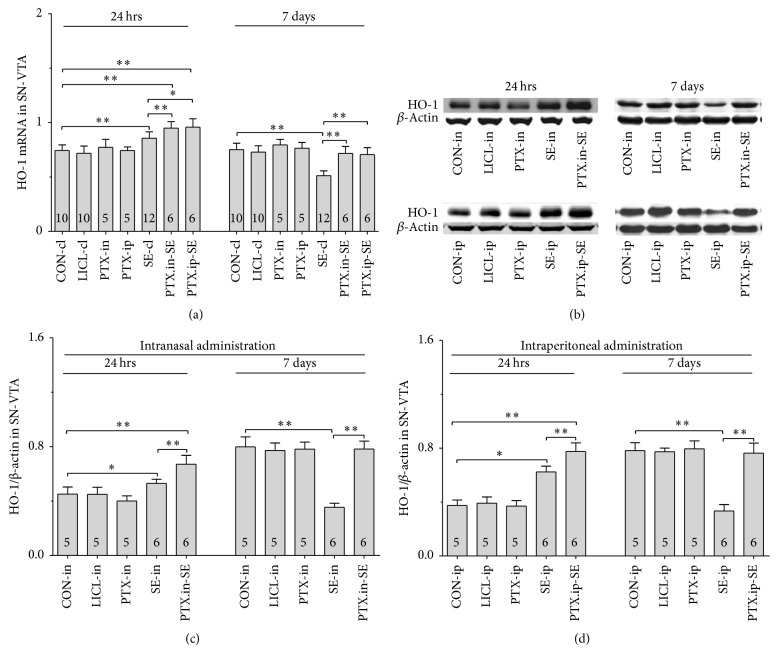
Effects of intranasal and intraperitoneal Ptx pretreatment on HO-1 in SN-VTA of SE rats induced by Li-Pc 24 hrs and 7 days after Pc injection. (a) HO-1 mRNA was detected by qPCR. (b–d) HO-1 protein was measured by Western blot. The results were expressed as the means ± SD. ^*∗*^*P* < 0.05; ^*∗∗*^*P* < 0.01.

**Figure 7:**
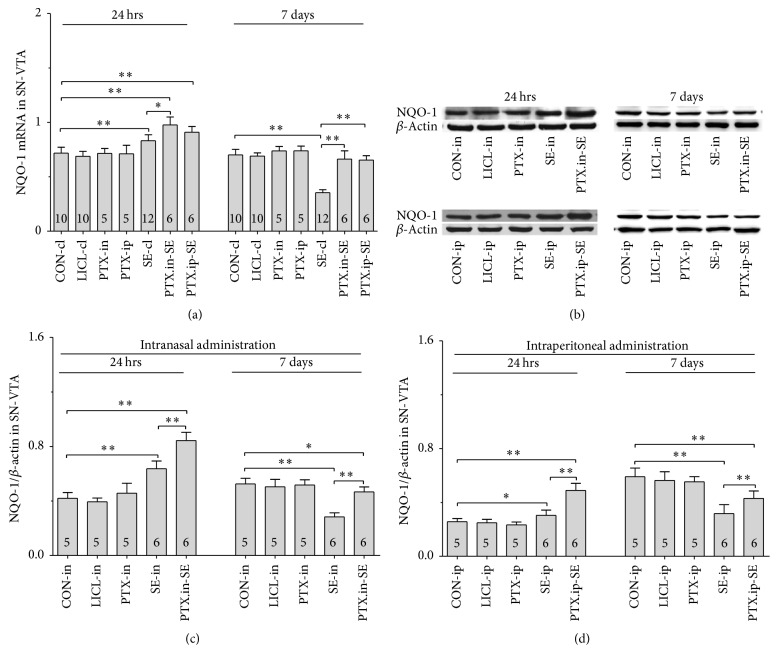
Effects of intranasal and intraperitoneal Ptx pretreatment on NQO-1 in SN-VTA of SE rats induced by Li-Pc 24 hrs and 7 days after Pc injection. (a–c) NQO-1 mRNA was detected by qPCR. (b–d) NQO-1 protein was measured by Western blot. The results were expressed as the means ± SD. ^*∗*^*P* < 0.05; ^*∗∗*^*P* < 0.01.

**Figure 8:**
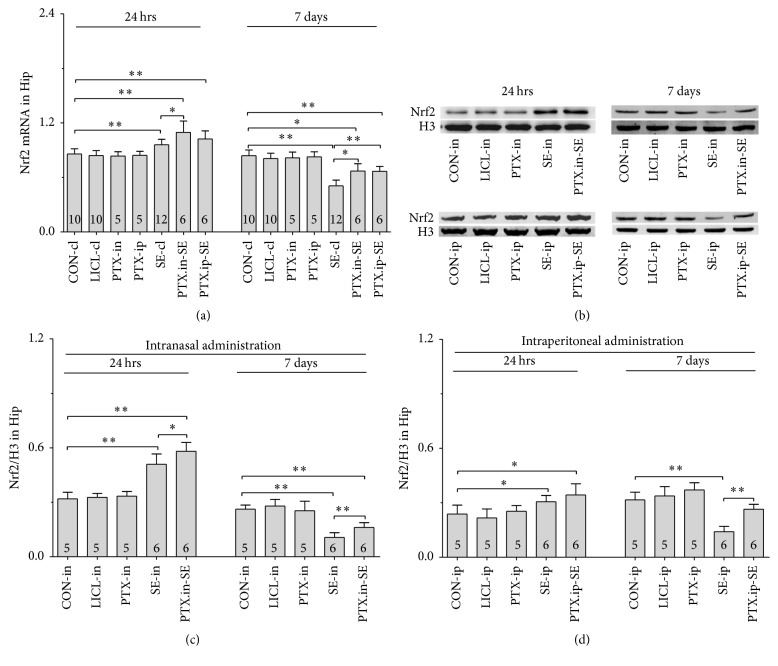
Effects of intranasal and intraperitoneal Ptx pretreatment on Nrf2 in Hip of SE rats induced by Li-Pc 24 hrs and 7 days after Pc injection. (a) Nrf2 mRNA was detected by qPCR. (b–d) Nrf2 protein was measured by Western blot. The results were expressed as the means ± SD. ^*∗*^*P* < 0.05; ^*∗∗*^*P* < 0.01.

**Figure 9:**
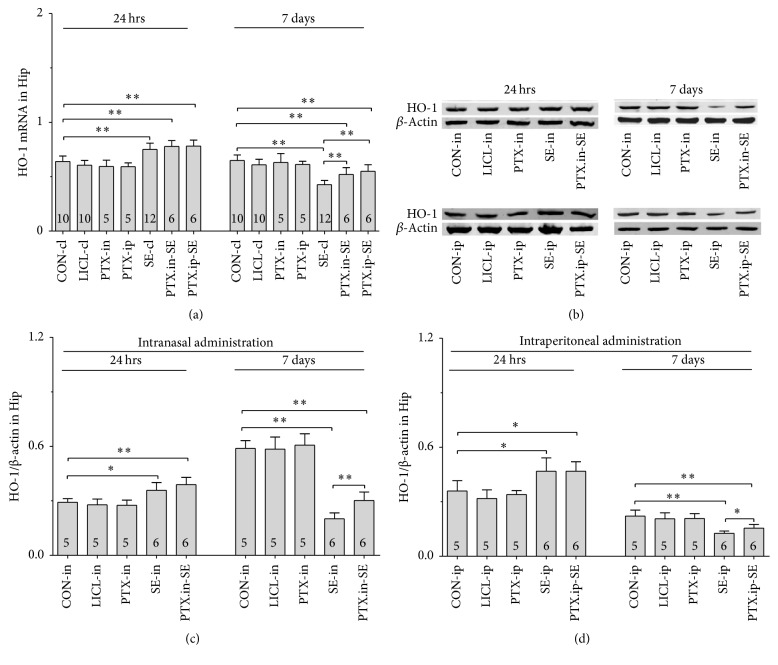
Effects of intranasal and intraperitoneal Ptx pretreatment on HO-1 in Hip of SE rats induced by Li-Pc 24 hrs and 7 days after Pc injection. (a) HO-1 mRNA was detected by qPCR. (b–d) HO-1 protein was measured by Western blot. The results were expressed as the means ± SD. ^*∗*^*P* < 0.05; ^*∗∗*^*P* < 0.01.

**Figure 10:**
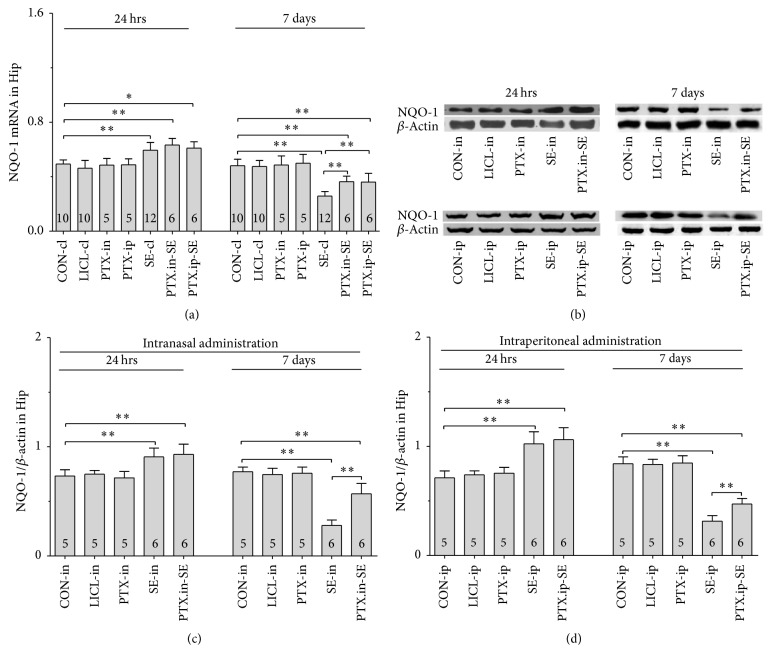
Effects of intranasal and intraperitoneal Ptx pretreatment on NQO-1 in Hip of SE rats induced by Li-Pc 24 hrs and 7 days after Pc injection. (a–c) NQO-1 mRNA was detected by qPCR. (b–d) NQO-1 protein was measured by Western blot. The results were expressed as the means ± SD. ^*∗*^*P* < 0.05; ^*∗∗*^*P* < 0.01.

Group differences among the CON-cl, LICL-cl, PTX-in, PTX-ip, SE-cl, PTX.in-SE, and PTX.ip-SE rats were found in the levels of Nrf2, HO-1, and NQO-1 mRNAs in SN-VTA (Figure 5(a), Nrf2 (24 hrs): *χ*^2^ = 36.650 and *P* < 0.01; Nrf2 (7 days): *χ*^2^ = 34.573 and *P* < 0.01; Figure 6(a), HO-1 (24 hrs): *χ*^2^ = 37.430 and *P* < 0.01; HO-1 (7 days): *F*(6,47) = 28.400 and *P* < 0.01; Figure 7(a), NQO-1 (24 hrs): *F*(6,47) = 27.723 and *P* < 0.01; NQO-1 (7 days): *χ*^2^ = 35.171 and *P* < 0.01), and Hip (Figure 8(a), Nrf2 (24 hrs): *χ*^2^ = 35.997 and *P* < 0.01; Nrf2 (7 days): *F*(6,47) = 37.925 and *P* < 0.01; Figure 9(a), HO-1 (24 hrs): *χ*^2^ = 39.571 and *P* < 0.01; HO-1 (7 days): *χ*^2^ = 36.662 and *P* < 0.01; Figure 10(a), NQO-1 (24 hrs): *F*(6,47) = 15.841 and *P* < 0.01; NQO-1 (7 days): *χ*^2^ = 40.301 and *P* < 0.01). The post hoc test revealed the elevation of Nrf2, HO-1, and NQO-1 mRNAs in SN-VTA and in Hip of SE-cl rats 24 hrs following Pc injection (*P* < 0.01) and the significant reduction of them 7 days after Pc administration (*P* < 0.01), compared with CON-cl rats. In SN-VTA, the increased Nrf2, HO-1, and NQO-1 mRNAs were detected in PTX.in-SE and PTX.ip-SE rats (*P* < 0.05), compared with SE-cl rats, except NQO-1 mRNA in PTX.ip-SE rats 24 hrs following Pc injection (*P* = 0.234). In Hip, the elevated Nrf2, HO-1, and NQO-1 mRNAs were observed only in PTX.in-SE and PTX.ip-SE rats 7 days following Pc injection, compared with SE-cl rats (*P* < 0.05).

#### 3.5.2. Nrf2, HO-1, and NQO-1 Proteins

The protein levels of Nrf2, HO-1, and NQO-1 extracted from SN-VTA and Hip were detected by Western blot. Nrf2, HO-1, and NQO-1 were located at approximately 110, 32, and 31 kDa, respectively. The group differences among CON-in, LICL-in, PTX-in, SE-in, and PTX.in-SE rats were found in the expression of Nrf2, HO-1, and NQO-1 in SN-VTA (Figures 5(b) and 5(c), Nrf2 (24 hrs): *F*(4,22) = 15.882 and *P* < 0.01; Nrf2 (7 days): *F*(4,22) = 84.169 and *P* < 0.01; Figures 6(b) and 6(c), HO-1 (24 hrs): *χ*^2^ = 21.011 and *P* < 0.01; HO-1 (7 days): *F*(4,22) = 71.535 and *P* < 0.01; Figures 7(b) and 7(c), NQO-1 (24 hrs): *χ*^2^ = 21.705 and *P* < 0.01; NQO-1 (7 days): *χ*^2^ = 17.290 and *P* < 0.01), and Hip (Figures 8(b) and 8(c), Nrf2 (24 hrs): *χ*^2^ = 20.481 and *P* < 0.01; Nrf2 (7 days): *F*(4,22) = 27.035 and *P* < 0.01. Figures 9(b) and 9(c), HO-1 (24 hrs): *χ*^2^ = 19.504 and *P* < 0.01; HO-1 (7 days): *F*(4,22) = 78.424 and *P* < 0.01; Figures 10(b) and 10(c), NQO-1 (24 hrs): *χ*^2^ = 17.695 and *P* < 0.01; NQO-1 (7 days): *F*(4,22) = 67.737 and *P* < 0.01), as well as in the levels of Nrf2 (Figures 5(b) and 5(d), SN-VTA; Figures 8(b) and 8(d), Hip; *P* < 0.01), HO-1 (Figures 6(b) and 6(d), SN-VTA; Figures 9(b) and 9(d), Hip; *P* < 0.01), and NQO-1 (Figures 7(b) and 7(d), SN-VTA; Figures 10(b) and 10(d), Hip; *P* < 0.01) among CON-ip, LICL-ip, PTX-ip, SE-ip, and PTX.ip-SE rats. The post hoc test showed that the expression of Nrf2, HO-1, and NQO-1 significantly increased in SN-VTA and in Hip of SE-in and SE-ip rats 24 hrs following Pc injection (*P* < 0.05) and then significantly decreased 7 days after Pc administration (*P* < 0.01), compared with corresponding control rats, respectively. In SN-VTA, Ptx pretreatment either by intranasal or intraperitoneal administration to SE rats elevated the levels of Nrf2, HO-1, and NQO-1 (*P* < 0.05). In Hip, except Nrf2 in PTX.in-SE (*P* < 0.05), the increased levels of Nrf2, HO-1, and NQO-1 were not found in PTX.in-SE and in PTX.ip-SE rats 24 hrs following Pc treatment, compared with SE rats. Seven days following Pc administration, the levels of Nrf2, HO-1, and NQO-1 of Hip were higher in Ptx-pretreated rats than in SE rats (*P* < 0.05). Nrf2, HO-1, and NQO-1 were involved in the attenuation of oxidative damage by intranasal Ptx pretreatment to SE rats.

## 4. Discussion

The present studies revealed that the epileptic seizures, the impaired visuospatial memory, and the defected mesodopaminergic system in SE rats induced by Li-Pc were effectively ameliorated by pretreatment of Ptx via intranasal delivery, as well as via intraperitoneal injection. Ptx pretreatment decreased the levels of LPO and MDA, increased the ratio of GSH/GSSG, and enhanced the transient activation of Nrf2 in SE rats. The enhanced transient activation of Nrf2 was followed by the upregulated HO-1 and NQO-1 in Ptx-pretreated SE rats. Compared with intraperitoneal injection, the poor visuospatial memory and the reduced levels of DA and its metabolites as well as TH and DAT were completely restored to normal levels by intranasal pretreatment of Ptx to SE rats. The results above demonstrated the amelioration of epileptic seizures, the alleviation of oxidative damage, and the involvement of Nrf2-ARE pathway in SE rats pretreated by intranasal Ptx.

Lithium-pilocarpine is used to induce SE to reproduce the most features of human SE [25, 26]. The SE rodent model induced by Li-Pc is one of the suitable experimental models in analyzing the pathophysiology of SE [25, 26, 34, 35]. Pilocarpine alone at higher doses not only results in a greater likelihood of induction of SE but also increases mortality rate [36, 37]; however, lithium chloride preadministered within 24 hrs effectively potentiates the epileptogenic action of Pc and simultaneously reduces the mortality of animals [38, 39]. The previous study found that, 30 minutes prior to induction of SE, intraperitoneal injection of Ptx at the dose of 60 mg/kg ameliorated the epileptic seizures best among the dose of 0, 20, 40, and 60 mg/kg [26]. Thus, 20 mg/kg Pc and 60 mg/kg Ptx were used in the present studies. Consistent with the previous studies [25, 26], it was found that all the rats treated by Li-Pc alone exhibited SE, the impaired visuospatial memory, the defected mesodopaminergic system, and the oxidative damage. Intranasal Ptx pretreatment to SE rats significantly delayed the epileptic seizures, ameliorated the deficits in visuospatial memory, restored the mesodopaminergic function, and attenuated the oxidative stress, which indicated that Ptx pretreatment to SE rats had neuroprotective effects.

Oxidative stress is one of the major factors detrimental to neurons in seizures [16, 40]. Epileptic seizures decrease the antioxidant capability and increase oxidative damage [16, 40, 41]. Redox status is a sensitive index of intracellular oxidative stress. GSH/GSSG is a biomarker of redox status in biological systems. We analyzed the ratio of GSH/GSSG by measuring GSH and GSSG in SN-VTA and Hip. GSH, the reduced form of glutathione, was significantly decreased and GSSG, the oxidized form of glutathione, was increased in SN-VTA and Hip of SE rats. GSH and GSSG are redox couples. Glutathione peroxidase catalyzes the reduction of two peroxide molecules using GSH to produce GSSG and water [42]. The diminution of GSH and elevation of GSSG as well as the decreased ratio of GSH/GSSG demonstrated the changes of redox status in SN-VTA and Hip of SE rats. Furthermore, two important lipid peroxidation markers, LPO and MDA, were detected in our studies. LPO and MDA are the products of the peroxidation of lipoproteins and phospholipids of biological membranes [43]. The present studies detected the high levels of LPO and MDA in SN-VTA and Hip of SE rats, which suggested that there was oxidative damage to cells in these brain regions of SE rats. Oxidative damage to cells in both Hip and SN-VTA might underlie the poor visuospatial memory of SE rats. Compared with SE rats, the reduced LPO and MDA as well as the increased ratio of GSH/GSSG suggested the higher antioxidative ability in SN-VTA and in Hip of PTX.in-SE rats. Increased antioxidative ability might be related to Nrf2-ARE pathway, one of important antioxidative defense systems in suppressing oxidative damage to neurons [44–47].

Nrf2 is a transcription factor and constitutes the main oxidative stress response in cells [13, 48]. Under the physiology condition, Nrf2 is located in the cytoplasm. When Nrf2 is activated, it is translocated into the nucleus and combined with the ARE to induce a series of cytoprotective enzymes such as HO-1 [49, 50] and NQO-1 [51, 52] to enhance the antioxidant capacity of cells and protect cells from the oxidative injury [53, 54]. In the present studies, the elevation of Nrf2, HO-1, and NQO-1 at mRNA and protein levels found in SE rats 24 hrs but not 7 days following Li-Pc injection indicated that epileptic seizures could transiently activate Nrf2-ARE pathway [55]. The transient activation of Nrf2-ARE pathway might be a response to the oxidative stress caused by SE [55], but SE-induced Nrf2 activation was not enough to protect neurons from oxidative damage, which underlay the defects in mesodopaminergic system, as well as the elevated LPO and MDA in SN-VTA and Hip of SE rats in the present studies. However, Nrf2-ARE pathway provides a potential target in controlling epileptic seizures [24, 55].

The relevance of Nrf2 in mesolimbic dopaminergic system has not been fully studied; however, it is known that Nrf2 plays more important role in maintaining normal nigral dopaminergic activity and protects the nigral dopaminergic neurons from neurodegeneration by reducing oxidative stress in Parkinson's disease [56–58]. Activation of Nrf2-ARE pathway reduces MPTP-induced neurotoxicity and the death of nigral dopaminergic neurons to a certain extent [46, 57]. Nigral dopaminergic neurons are more susceptible to MPTP-induced damage in Nrf2−/− mice than Nrf2+/+ mice [56–58]. Compared with SE rats, the significant elevation of Nrf2, HO-1, and NQO-1 in SN-VTA and Nrf2 in Hip of intranasal Ptx-pretreated SE rats 24 hrs following Pc injection indicated the region-specific involvement of Nrf2-ARE pathway. The following reasons might account for the discrepancy between SN-VTA and Hip. The first might be the fact that Nrf2 differed between various neuronal subpopulations and regulated different gene products in the nigral neurons versus hippocampal neurons [59]. The second reason might be related to DA. The neurotransmitter DA itself can be a source of oxidative stress. Prior to SE, dopaminergic neurons themselves experience the oxidative stimulus due to autooxidation of DA [60, 61].

Pentoxifylline, as a nonspecific phosphodiesterase inhibitor, might exert its pharmacological effects during the acute phase of SE by decreasing inflammatory cytokine production [62, 63]. Moreover, based on our observations on rats treated by Ptx alone, Ptx did not work as Nrf2 activator, which was consistent with the results published by Ahmed and El-Awdan [64]. Administration of Ptx to rats did not alter Nrf2 expression [64]. Evidences demonstrate that the protective role of Nrf2-ARE pathway is mediated by HO-1 and NQO-1 [24]. Compared with control rats or SE rats 24 hrs following Pc, we found that intranasal Ptx pretreatment to SE rats significantly increased the levels of Nrf2, followed by upregulation of HO-1 and NQO-1 at mRNA and protein levels in Ptx-pretreated SE rats, which suggested that intranasal administration of Ptx might increase the antioxidative capability of cells by enhancing the SE-induced transient activation of Nrf2. Due to the fact that the half-life of Nrf2 is very short [65] and at 24 hrs from SE it could be degraded, the time points we choose were 24 hrs and 7 days after administration of Pc as they encompassed both the acute and latent epileptic conditions [66]. How Nrf2 was enhanced in SE rats by Ptx pretreatment might not be determined 24 hrs following Pc injection. Earlier time points in Nrf2 induction of Ptx-pretreated SE rats following Pc injection are necessary to actually unravel if Ptx is cooperating with SE to induce Nrf2 in the future studies.

The large surface area of the nasal mucosa and the abundant blood supply of the nasal cavity make intranasal administration a viable option for delivery of diverse therapeutic compounds [67, 68]. The unique anatomical connection between the nasal cavity and the brain provides direct nose-to-brain delivery of drugs to target the brain through pathways along the olfactory and trigeminal nerves innervating the nasal cavity [5, 7]. The restoration of impaired visuospatial memory and mesodopaminergic activity in PTX.in-SE rats indicated that intranasal administration of Ptx effectively targets the brain. Compared with PTX.ip-SE rats, the parameters in visuospatial memory and mesodopaminergic system observed in PTX.in-SE rats were completely recovered to the level of control rats 7 days after Pc injection; however, the partial parameters in visuospatial memory and mesodopaminergic system were still lower in PTX.ip-SE rats than in controls. Intranasal administration of Ptx seemed more efficient in controlling SE than intraperitoneal injection. In addition to nose-to-blood-to-brain route (an extensive nasal absorption of the Ptx to the bloodstream), direct nose-to-brain route might be involved in targeting a substantial fraction of Ptx to the brain. Furthermore, Ptx administered intranasally might be reaching the brain expectedly much earlier than through intraperitoneal administration prior to coming insults. Whether a direct transport of Ptx was occurring from nose to the brain, Ptx concentration levels in different cerebral regions and the corresponding rostral-to-caudal biodistribution patterns should be determined following both routes of administration in the future studies.

In conclusion, intranasal delivery of Ptx to rats significantly suppressed the epileptic seizures induced by Li-Pc, ameliorated the deficits in visuospatial memory and in mesodopaminergic system, and enhanced the transient activation of Nrf2 in SE rats. Intranasal administration of Ptx could effectively protect cells from oxidative damage in SE and may hopefully become a noninvasive, painless, and easily administered option for epileptic patients.

## Figures and Tables

**Table 1:** Validation parameters of the LC-MS/MS method.

Analyte	*r*	LLOQ (ng/g)	Recovery (%)	Intraprecision (RSD%)	Interprecision (RSD%)
DA	0.9969	2.0	94.6 ± 8.7	10.3	12.4
DOPAC	0.9982	72.0	93.4 ± 6.6	8.9	7.5
HVA	0.9977	50.0	95.3 ± 7.4	7.7	11.3

DA: dopamine; DOPAC: 3,4-dihydroxyphenylacetic acid; HVA: homovanillic acid.

**Table 2:** Effects of pentoxifylline pretreatment on status epilepticus rats.

Group	*n*	Seizures (%)	Latency to first seizure (min)	SE (%)	Mortality (%) within 24 h
CON-cl	50	0 (0)	—	—	0
LICL-cl	50	0 (0)	—	—	0
PTX-in	25	0 (0)	—	—	0
PTX-ip	25	0 (0)	—	—	0
SE-cl	70	100	13.87 ± 4.73	100	10
PTX.in-SE	35	17.14^*∗*^	37.78 ± 6.25^*∗*^	0	0
PTX.ip-SE	35	20^*∗*^	28.08 ± 7.10^*∗*^	0	0

^*∗*^ *P* < 0.01 versus SE group.

**Table 3:** Effects of pentoxifylline pretreatment on DA and its metabolites of status epilepticus rats.

	CON-cl	LICL-cl	PTX-in	PTX-ip	SE-cl	PTX.in-SE	PTX.ip-SE
CPu							
DA (ng/g)	223.4 ± 11.9	210.8 ± 13.6	233.2 ± 15.0	234.6 ± 10.0	121.0 ± 11.0^*∗∗*^	202.9 ± 20.4^#^	209.5 ± 21.5^#^
	(5.3)	(6.4)	(6.4)	(4.3)	(9.1)	(10.1)	(10.3)
DOPAC (ng/g)	3437.5 ± 196.2	3264.2 ± 94.9	3716.2 ± 221.2	3634.9 ± 251.4	2355.7 ± 182.9^*∗∗*^	3231.0 ± 302.5^#^	3271.0 ± 224.1^#^
	(5.7)	(2.9)	(6.0)	(6.9)	(7.8)	(9.4)	(6.9)
HVA (ng/g)	879.2 ± 50.0	852.4 ± 60.1	889.1 ± 75.0	901.1 ± 33.9	462.1 ± 47.1^*∗∗*^	791.0 ± 88.7^#^	842.7 ± 54.7^#^
	(5.7)	(7.1)	(8.4)	(3.8)	(10.2)	(11.2)	(6.5)
Hip							
DA (ng/g)	10.7 ± 0.6	10.2 ± 0.8	11.2 ± 0.9	11.4 ± 1.2	5.1 ± 0.6^*∗∗*^	9.7 ± 0.8^#^	9.4 ± 0.6^*∗*#^
	(5.9)	(7.4)	(8.4)	(10.5)	(12.1)	(8.5)	(6.5)
DOPAC (ng/g)	365.7 ± 29.1	352.0 ± 20.5	383.7 ± 38.7	380.8 ± 23.4	116.5 ± 14.9^*∗∗*^	340.8 ± 25.4^#^	325.5 ± 29.7^#^
	(8.0)	(5.8)	(10.0)	(6.1)	(12.8)	(7.5)	(9.1)
HVA (ng/g)	106.5 ± 7.3	98.3 ± 5.8	111.9 ± 8.8	107.6 ± 7.4	64.8 ± 6.0^*∗∗*^	100.2 ± 5.1^#^	96.4 ± 6.9^#^
	(6.8)	(5.9)	(7.9)	(6.8)	(9.3)	(5.1)	(7.2)

CPu: caudate putamen; Hip: hippocampus; DA: dopamine; DOPAC: 3,4-dihydroxyphenylacetic acid; HVA: homovanillic acid. The value in brackets is the coefficient of variation.

^*∗*^ *P* < 0.05 and ^*∗∗*^*P* < 0.01 versus CON-cl; ^#^*P* < 0.01 versus SE-cl.
